# Rare spontaneous formation of a dentin bridge post-dental trauma: A cone beam computed tomography-based case report with root canal therapy

**DOI:** 10.1016/j.radcr.2025.11.082

**Published:** 2026-01-05

**Authors:** Moslem Rostami, Hengameh Ashraf, Yaser Safi, Bita Heydarzadeh

**Affiliations:** a Department of Endodontics, School of Dentistry, Shahid Beheshti University of Medical Sciences, Tehran, Iran; b Department of Oral and Maxillofacial Radiology, School of Dentistry, Shahid Beheshti University of Medical Sciences, Tehran, Iran

**Keywords:** Cone beam computed tomography, Dental trauma, Dentin bridge, Tertiary dentin, Odontoblast

## Abstract

Spontaneous dentin bridge formation in mature teeth without pulp exposure or therapeutic intervention is an exceedingly rare phenomenon. This case report describes a 54-year-old male patient presenting with gray discoloration of the right maxillary central incisor (tooth #11) following dental trauma in 2019. Clinical examination indicated coronal pulp necrosis, while periapical radiography and cone-beam computed tomography (CBCT) confirmed internal resorption in the coronal pulp chamber with a thick, regular dentin bridge formed spontaneously beneath the resorbed area. The dentin bridge preserved the vitality of the apical pulp, as evidenced by a normal apical root canal. Treatment involved root canal therapy limited to the necrotic coronal pulp up to the dentin bridge, followed by internal bleaching to improve tooth color and esthetic restoration with composite resin. A 3-month follow-up radiograph showed treatment stability with no pathological changes. This case highlights the reparative potential of vital apical pulp in traumatized teeth and the critical role of cone-beam computed tomography (CBCT) in diagnosing such rare occurrences. However, the absence of histological confirmation and short follow-up duration limit conclusions on long-term stability.

## Introduction

Dental trauma is among the most common oral injuries, frequently affecting the maxillary anterior teeth, and may lead to crown fractures, pulp damage, or tooth loss [1]. The dentin bridge, a layer of hard dentinal tissue produced by odontoblast-like cells, was first described in 1938 by Zander [2]. This structure forms through the secretion of dentinal matrix by activated odontoblasts in response to pulp stimulation, such as trauma or bioactive materials [3].

In protective pulp treatments, such as direct pulp capping with calcium-silicate cements, dentin bridge formation is reported in 85%-90% of cases [4]. In cases of pulp exposure, materials like calcium hydroxide or mineral trioxide aggregate (MTA) can stimulate dentin bridge formation [5].

Internal resorption is another, less common condition that may occasionally be associated with dentin bridge formation. This pathological process, driven by clastic cells in inflamed pulp, can progress to pulp necrosis [6]. However, if vital odontoblast-like cells remain in the apical region or around the resorption lacuna, reparative hard tissue may form, helping to preserve pulp vitality [7].

Spontaneous dentin bridge formation beneath the site of internal resorption in mature teeth without pulp exposure or treatment is an extremely rare phenomenon, reported only in a few cases [8,9]. In this rare case, we will discuss the spontaneous formation of regular and thick dentin bridge in the coronal pulp region following dental trauma without any pulp exposure. This report presents such a case following dental trauma, emphasizing cone-beam computed tomography’s (CBCT) diagnostic superiority over conventional radiography.

## Case report

**Fig. 1:**
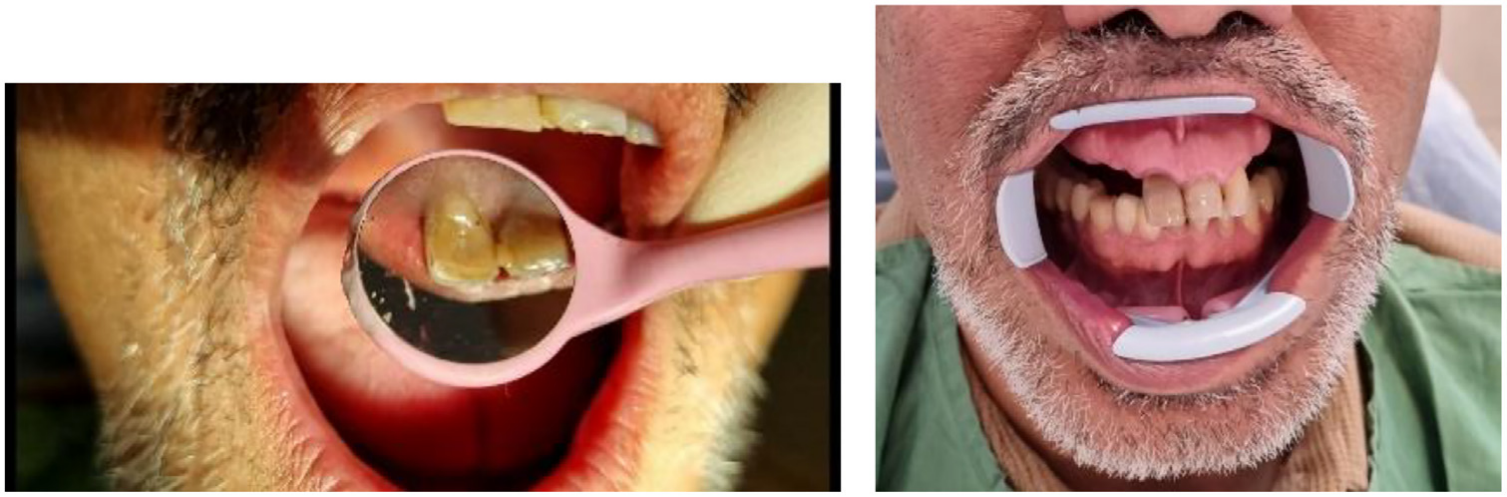
Initial photographies.

A 54-year-old smoker male patient with no systemic diseases presented to the Shahid Beheshti University School of Dentistry with a chief complaint of gray discoloration and esthetic concerns regarding the right maxillary central incisor (tooth #11) (Fig. 1). The patient reported a history of nasopalatine cyst surgery (1 year ago) and direct trauma to the maxillary anterior teeth in the summer of 2019. He denied pain, swelling, or tooth mobility.

**Fig. 2:**
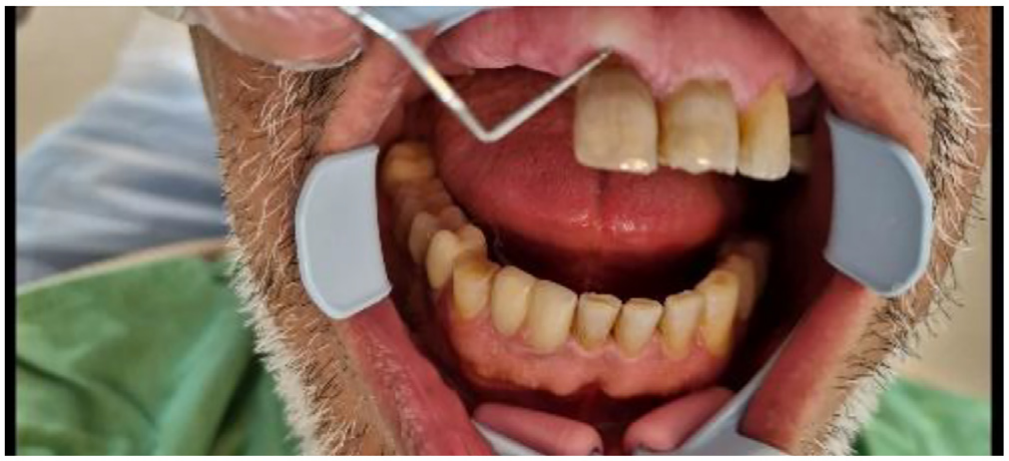
Probing test.

**Fig. 3:**
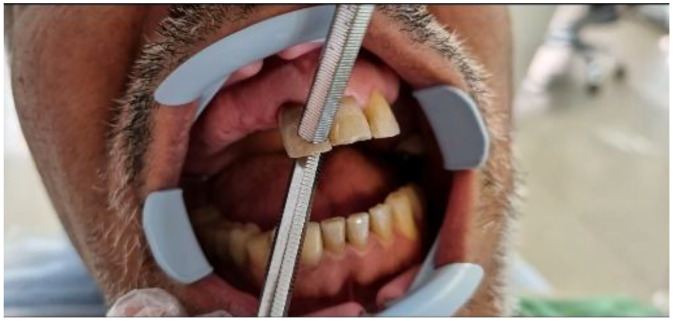
Mobility test.

Clinical examination revealed normal probing depths (2-3 mm) (Fig. 2) and negative mobility (Fig. 3). Sensibility test including cold (ice stick, −50°C), heat (heated gutta-percha), and electric pulp testing (EPT) were negative. All tests were repeated after a 5-minute interval for confirmation. Positive percussion and negative palpation tests for tooth #11, are suggestive of coronal pulp necrosis. A small distal composite restoration, approximately 2 mm from the pulp, was noted, with no evidence of fracture or pulp exposure (Fig. 1). Intraoral and extraoral examinations showed no swelling or asymmetry.

**Fig. 4:**
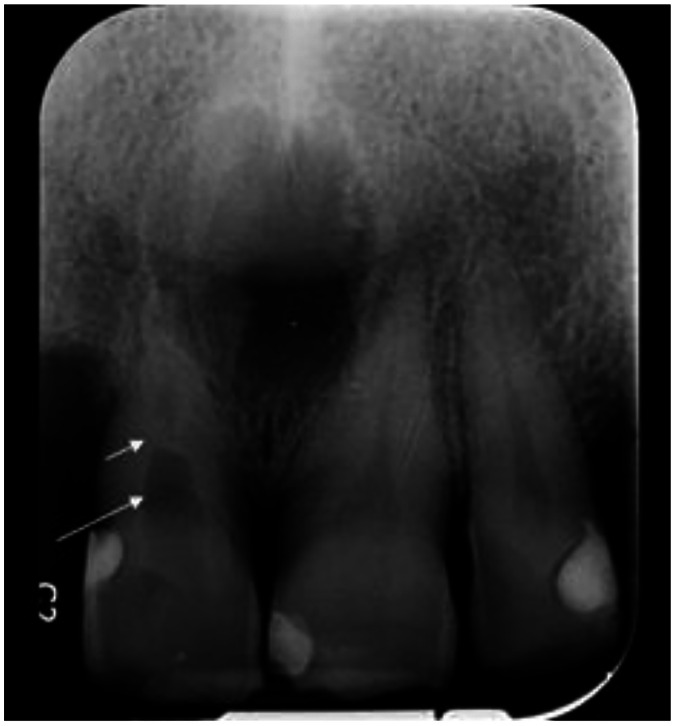
Initial Periapical radiography. Dentin bridge (short arrow), internal root resorption (long arrow).

Periapical radiography revealed internal resorption in the cervical region of tooth #11, with a dentin bridge formed spontaneously below the resorbed area and a normal root canal distally (Fig. 4). Evidence of prior nasopalatine cyst surgery with well-defined borders was also observed.

**Fig. 5:**
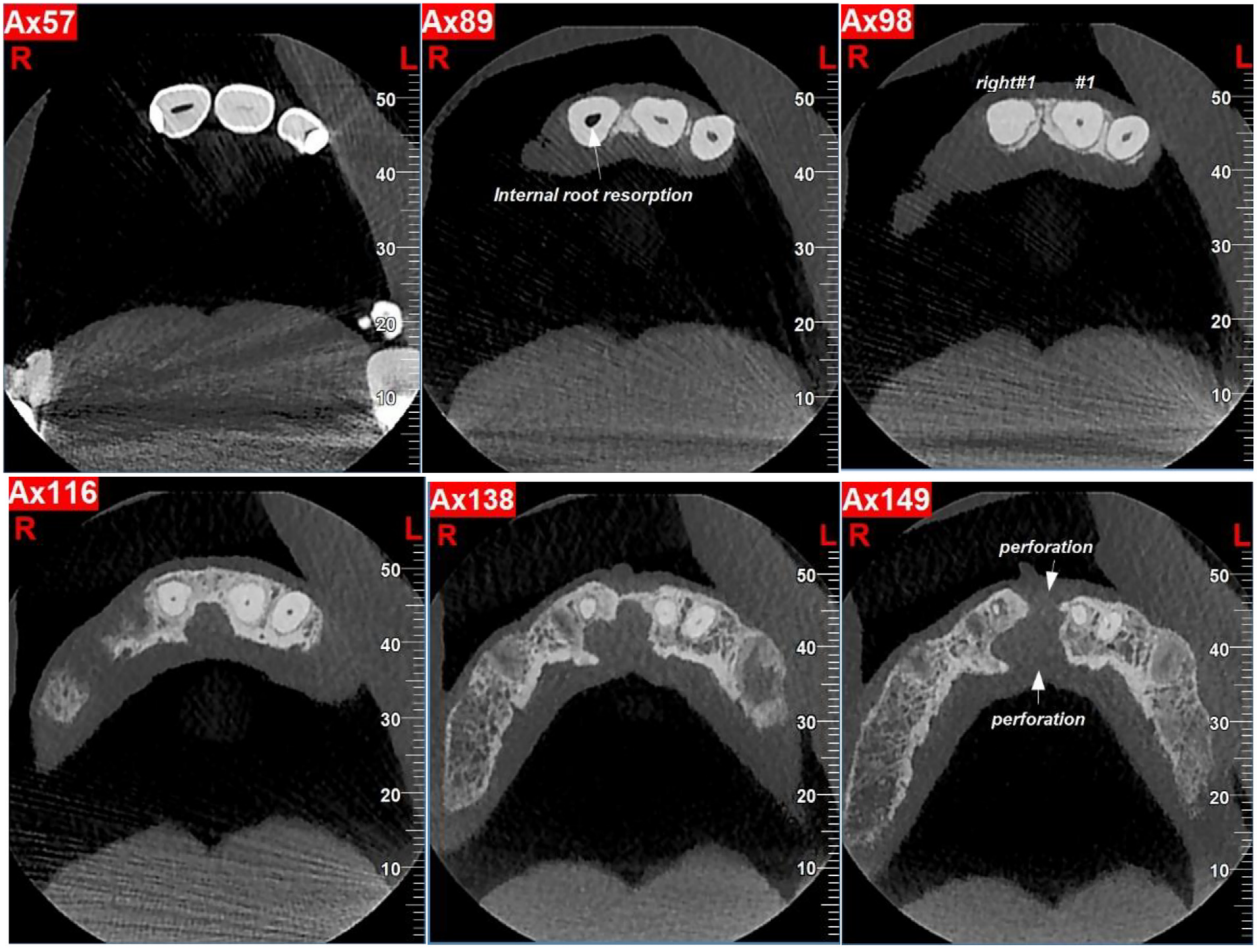
Occlusal views of CBCT images show internal root resorption, bone loss and perforation of cortical plates.

**Fig. 6:**
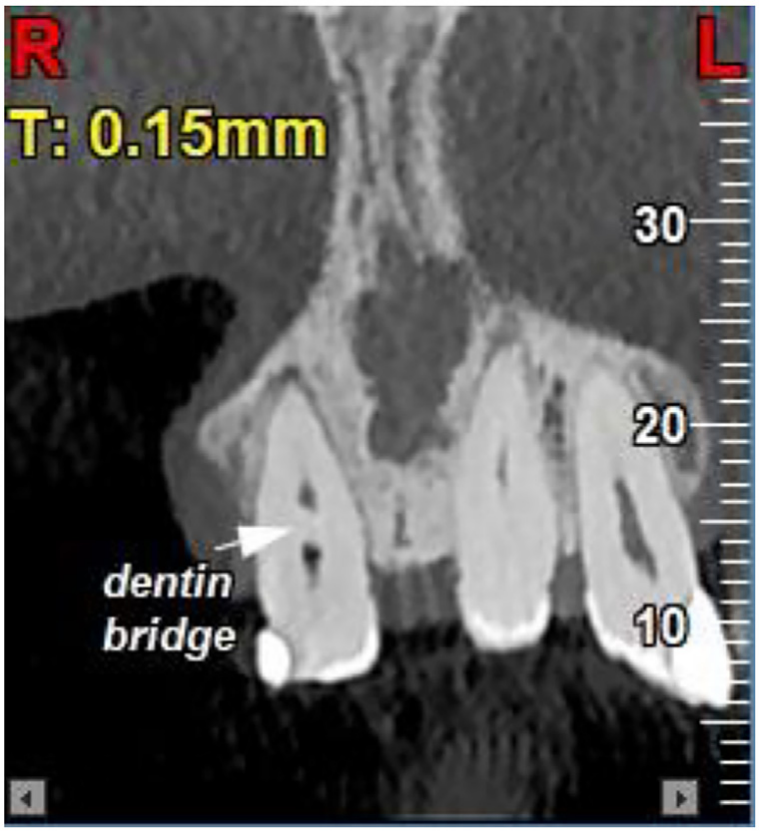
Coronal view of CBCT image shows dentin bridge separating the necrotic coronal pulp from the vital apical pulp.

**Fig. 7:**
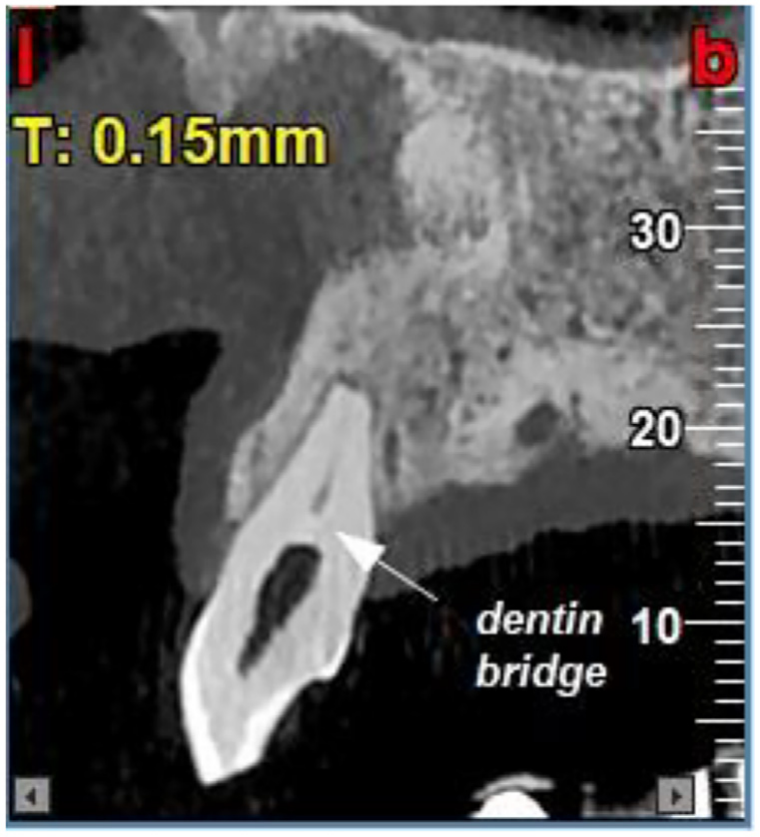
Sagittal view of CBCT image shows dentin bridge beneath the area of internal root resorption.

Conventional periapical radiography could not reliably demonstrate the presence, thickness, or continuity of a dentin bridge or the exact extent of resorption. Therefore, to confirm the diagnosis, a cone-beam computed tomography (NewTom VGi evo) scan with a 6×6 cm field of view (FOV) in high resolution and voxel size of 0.15 mm was obtained (Fig. 5, Fig. 6, Fig. 7). The CBCT confirmed internal resorption in the coronal pulp chamber, a thick and regular dentin bridge beneath the resorption site, and a normal apical root canal. Widening of the periodontal ligament (PDL) was noted, along with buccal and lingual cortical perforations from the previous cyst surgery. No fracture or pulp exposure was observed. The coronal pulp was necrotic, while the pulp below the dentin bridge remained vital, likely due to the protective dentin bridge formed by healthy odontoblasts in the apical region [6,7]. Spontaneous dentin bridge formation beneath internal resorption in mature teeth without treatment is exceedingly rare [8,9].

**Fig. 8:**
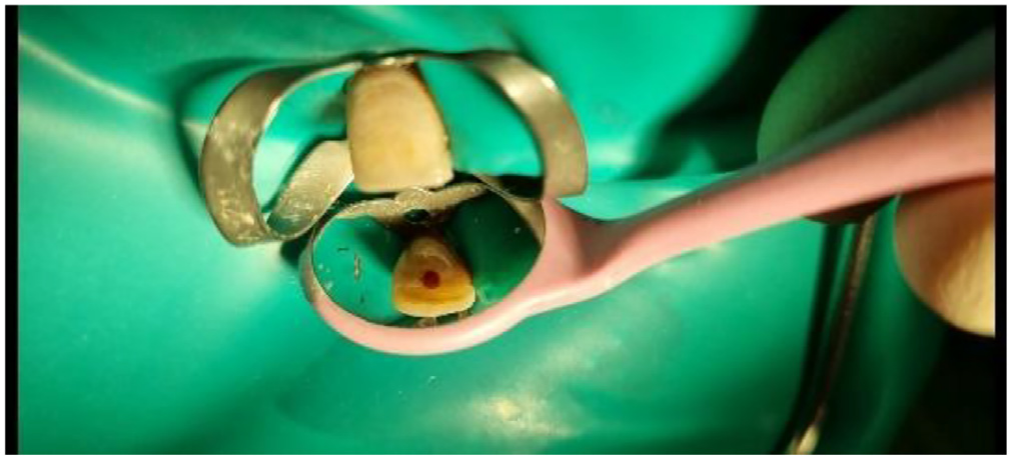
Photography of access cavity preparation.

**Fig. 9:**
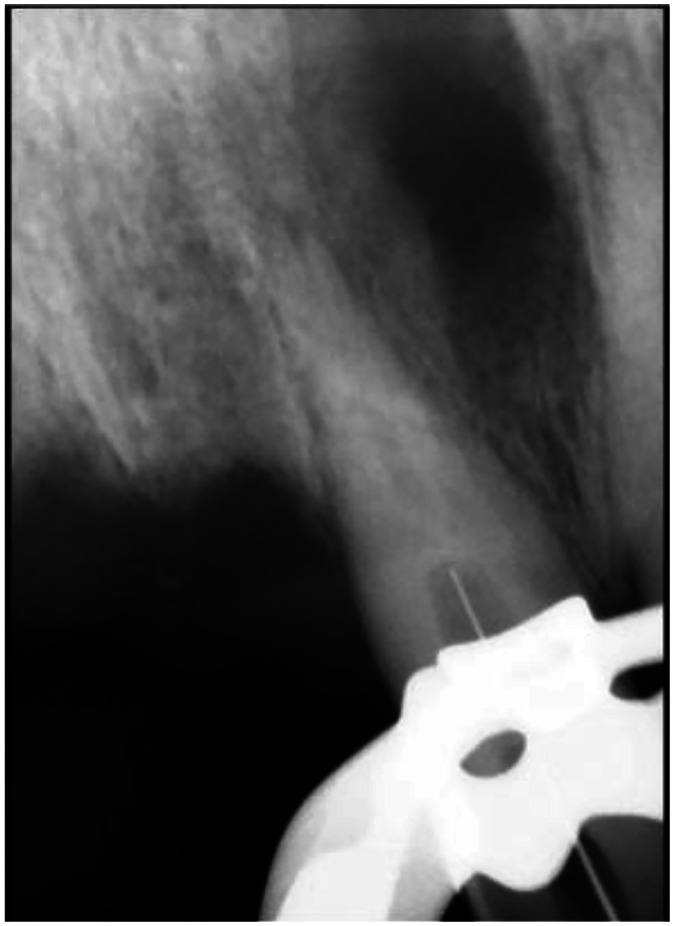
Periapical radiography during treatment.

**Fig. 10:**
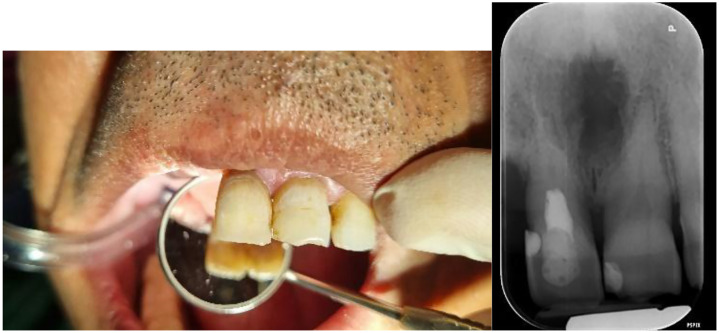
Periapical radiography and photography after root canal therapy and tooth restoration.

Treatment involved root canal therapy up to the level of the dentin bridge using warm vertical technique, followed by internal bleaching to enhance the tooth's color. The access cavity was subsequently restored with composite resin (Figs. 8 and 9). The initial access cavity revealed the necrotic coronal pulp (Fig. 8). Periapical radiography during treatment confirmed working length and canal obturation (Fig. 9). Finally, the tooth was restored on access cavity after internal bleaching (Fig. 10).

**Fig. 11:**
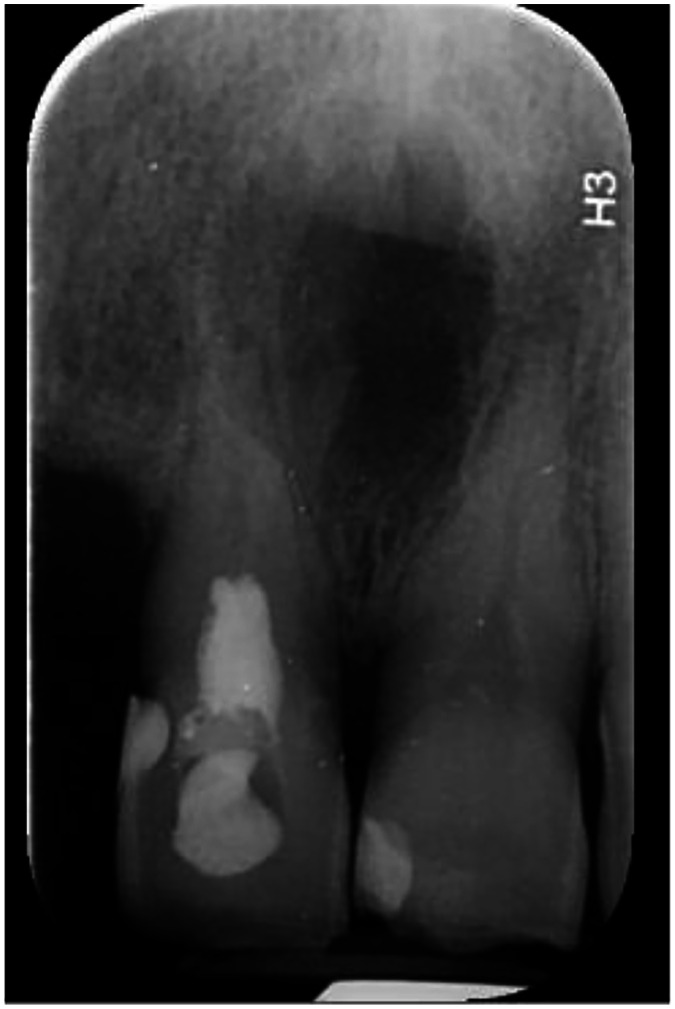
Three-month follow-up Periapical radiography.

A 3-month follow-up radiograph demonstrated treatment stability with no pathological changes, and the patient reported no symptoms (Fig. 11).

Written informed consent was obtained from the patient for publication. This case highlights the rare occurrence of spontaneous dentin bridge formation beneath internal resorption, underscoring the reparative potential of vital apical pulp in traumatized teeth [7,8].

## Discussion

The spontaneous formation of a dentin bridge in the coronal pulp region following dental trauma, represents a rare reparative response in mature teeth without pulp exposure or therapeutic intervention. This finding suggests that the dental pulp may retain considerable regenerative capacity even when the coronal portion becomes necrotic, provided vital cells capable of hard-tissue formation remain in the apical region.

In the present case, a well-defined hard-tissue barrier was observed beneath the internal resorption, appearing to separate the necrotic coronal pulp from vital apical tissue. This barrier likely contributed to the preservation of apical pulp vitality, as evidenced by the absence of apical pathosis and a normal apical canal configuration on CBCT imaging.

Typically internal resorption, in inflamed pulp, is a known precursor to pulp necrosis if inflammation progresses unchecked [6]. However, limited forms of resorption can sometimes be associated with concurrent reparative dentinogenesis. A study by Ricucci et al. [2] highlighted that internal resorption, when limited and followed by reparative dentinogenesis, can lead to the formation of dentin-like structures, supporting pulp survival in traumatized teeth. This aligns with our findings, where the dentin bridge formed spontaneously below the resorption site, suggesting a localized reparative response to trauma-induced inflammation.

The role of dental trauma in triggering such a response is noteworthy. The patient’s history of direct trauma to the maxillary anterior teeth in 2019 likely initiated a cascade of inflammatory and reparative processes within the pulp. A study by Palcze et al. demonstrated that traumatic injuries can stimulate odontoblast activity, leading to tertiary dentin formation in the absence of pulp exposure, particularly in cases where the pulp retains some degree of vitality [3]. The spontaneous nature of the dentin bridge in this case, without the application of bioactive materials like mineral trioxide aggregate (MTA) or calcium hydroxide, further distinguishes it from typical pulp-capping scenarios, where dentin bridge formation is reported in 85%-90% of cases (4). Table 1 summarizes the marked difference in the frequency of dentin bridge formation between common clinical interventions and spontaneous cases, highlighting the exceptional rarity of the present report.

**Table 1:** Frequency of dentin bridge formation under different clinical conditions.

Clinical situation	Frequency of dentin bridge formation	References
Direct pulp capping (MTA, Biodentine, etc.)	High (85%-96%)	4,5
Indirect pulp capping	Frequent (approximately 70%-95%, highly material and technique dependent)	General endodontic literature
Spontaneous without pulp exposure or therapeutic intervention	Exceedingly rare	8, 9, and present study

Instead, this case aligns more closely with rare reports of spontaneous dentinogenesis, as documented by Patel and Khazane [8,9], though such occurrences remain exceptional in mature teeth. A summary of the cited related studies and their comparison with the present case is presented in Table 2.

**Table 2:** Summary of the related studies and their comparison with the present case.

Study	Age (years)	Tooth	Etiology	Location	Histology

Patel and Saberi [8]	32	#21	Trauma	Coronal	No
Khazane et al. [9]	28	#11	Caries	Mid-root	Yes
Tzanetakis [7]	19	#21	Intrusion	Apical	No
Present case	54	#11	Trauma	Coronal	No

The clinical implications of this case are significant. The presence of a dentin bridge allowed for conservative root canal therapy limited to the necrotic coronal pulp, preserving the vital apical pulp and avoiding more invasive procedures. This highlights the importance of advanced imaging, such as CBCT, in accurately diagnosing the extent of resorption and the vitality of the pulp, which directly informs treatment planning. A study by Dao et al. [10] emphasized the utility of CBCT in detecting dentin bridge formation and assessing pulp vitality in complex cases of internal resorption, supporting its role in guiding minimally invasive interventions. Furthermore, the esthetic restoration with composite resin addressed the patient’s primary concern, demonstrating that functional and esthetic outcomes can be achieved even in such rare cases.

## Clinical and imaging points

• Spontaneous dentin bridge can preserve apical vitality in traumatized teeth.
• CBCT (voxel ≤0.2 mm) is essential for diagnosis and treatment planning.
• Conservative RCT up to bridge is feasible.

## Limitations

• No histological confirmation of odontoblastic activity.
• Short follow-up (3 months)—long-term stability uncertain.
• Spontaneous bridges may be less mineralized than biomaterial-induced ones [9].

## Conclusion

This case demonstrates rare spontaneous dentin bridge formation post-trauma, enabling conservative treatment. CBCT was pivotal in diagnosis and management. Long-term studies are needed to assess durability of spontaneous bridges.

## Patient consent

The patient provided informed consent for his clinical information and images to be included in this case report. He understood the purpose of the publication, how his privacy would be protected, and agreed voluntarily to share his data for research purposes.
